# pH-Induced Electrostatic Interaction between Polyacrylates and Amino-Functionalized Graphene Oxide on Stability and Coating Performances

**DOI:** 10.3390/polym13193406

**Published:** 2021-10-03

**Authors:** Wenbo Zhang, Sichun Li, Jianzhong Ma, Yingke Wu, Chao Liu, Hongxia Yan

**Affiliations:** 1 Shaanxi Collaborative Innovation Center of Industrial Auxiliary Chemistry and Technology, Shaanxi University of Science and Technology, Xi’an 710021, China; lc1010158@163.com; 2 College of Chemistry and Chemical Engineering, Shaanxi University of Science and Technology, Xi’an 710021, China; L15191562651@163.com; 3 College of Bioresources Chemical and Materials Engineering, Shaanxi University of Science and Technology, Xi’an 710021, China; einske@163.com; 4 School of Chemistry and Chemical Engineering, Northwestern Polytechnical University, Xi’an 710129, China; hongxiayan@nwpu.edu.cn

**Keywords:** graphene oxide, polyacrylate, amino group, surface charge

## Abstract

Electrostatic interaction between polymers and nanofillers is of great importance for the properties and design of their composites. Polyacrylates with carboxyl, hydroxyl and acylamino groups were synthesized via emulsion polymerization and marked as P(MMA-BA-AA), P(MMA-BA-HEA) and P(MMA-BA-AM), respectively. Amino-functionalized graphene oxide (NGO) was prepared by Hoffman rearrangement using GO as the raw material. The polyacrylate composites were prepared by mixing NGO with each of the three kinds of polyacrylate. Effects of pH and NGO amounts on the properties of polyacrylate composites were studied. It was found that the surface charge of polyacrylate and NGO had the greatest effect on the composite properties. P(MMA-BA-AM)/NGO was not stable at any pH (2–8). With the same NGO amount of 0.1 wt%, the toughening effect of NGO on P(MMA-BA-AA) was larger than that on P(MMA-BA-HEA). The break strength of P(MMA-BA-AA)/NGO and P(MMA-BA-HEA)/NGO increased to 5.22 MPa by 47% and 3.08 MPa by 31%, respectively. NGO could increase the thermal stability of P(MMA-BA-AA) and P(MMA-BA-HEA) to different degrees. The polyacrylate film-forming processes were tested, and it showed that NGO influenced polyacrylate through the whole film-forming process. The results provide potential methods for the design of polymer-based nanocomposites.

## 1. Introduction

Polymer based graphene composites is an important application field because graphene could increase the properties of composites such as mechanical properties [1,2], electrical performances [3,4], thermal properties [5,6], barrier properties [7,8], flame retardancy [9,10], biomedical applications [11,12], etc. For example, Vasile et al. [13] functionalized graphene oxide with 2-hydroxyethyl methacrylate (HEMA) to prepare a composite with a poly(propylene fumarate)/poly(ethylene glycol) dimethacrylate (PPF/PEGDMA) matrix. The compressive strength, compressive modulus, morphology, equilibrium water content, degradation and mineralization behavior have been investigated and showed effective support for bone tissue formation. The performances of polymer composites changed greatly by graphene itself or by the synergistic effect of graphene and polymer.

Dispersion of graphene or graphene-based nanomaterials in polymers is a hot topic with which researchers are concerned. It is influenced by the interfacial adhesion or interaction between graphene and polymers. In order to improve the performance of composites, lots of methods have been designed and used. The most common method is to graft graphene on a target polymer by covalent bonding. Park et al. [14] conjugated aniline to few-layer graphene via perfluorophenyl azide (PFPA)-mediated coupling chemistry. A subsequent in situ polymerization of aniline gave polyaniline covalently grafted on the few-layer graphene surface. The method can be readily adapted to grow polyaniline or other conducting polymers on these technologically important carbon materials. In the report of Wu et al. [15], polyethylene glycol grafted graphene nanosheets (PEG-g-GO) and poly(vinylidene fluoride)/PEG-g-GO nanocomposites were synthesized by a solution mixing method. Polyethylene glycol, through increasing the insulation capacity and free volume, significantly increased the dielectric permittivity and led to a low loss for the composite.

Surfactants or substances that have good compatibility with both graphene and polymers can also be utilized to improve the interfacial adhesion. Firdaus et al. [16] added dodecylbenzene sulfonic acid (DBSA) surfactant in graphene/polyaniline nanocomposites, which developed a homogenous and uniform dispersion of the graphene filler within the polyaniline matrix. The electrical conductivity and thermal conductivity of the composites increased by 10% and 6%, respectively, compared with those without DBSA. Li et al. [17] used β-cyclodextrin (β-CD) as the bonding agent with its unique structure to form a supramolecular system with graphene oxide layers. They generated the inclusion compound with aniline monomers via in situ polymerization of polyaniline on the surface of graphene oxide. The obtained reduced graphene oxide/carbon nanodots/polyaniline nanocomposites (RGO@CN/PANI) had satisfied performance for supercapacitors.

Functionalizing graphene with organic chains or polymers that have good compatibility with the target polymer is another effective method. In Wang’s work [18], functionalized reduced GO (FRGO) was obtained by solvothermal reduction with sodium borohydride as the reduction agent and ammonia hydroxide as the nitrogen source. FRGO/PANI composites were achieved by synthesizing polyaniline (PANI) with FRGO as a substrate via in situ polymerization. The introduction of amino groups facilitates π–π conjugation and covalent bonding between FRGO sheets and PANI chains, resulting in an intimate and uniform contact between FRGO sheets and PANI chains. Wang et al. [19] chemically modified GO with cystamine dihydrochloride (CDHC), leading to a tight interaction between graphene and rubber owing to the disulfide bonds in CDHC molecules, which can participate in the vulcanization reaction of rubber through disulfide exchange during thermal processing.

For waterborne polymers, especially for polyacrylate, physical blending is a simple and efficient process to introduce graphene or graphene-based nanomaterials. However, simple blending without further interaction could not improve the properties of the composite to a higher level. In our previous work [20], amino-functionalized graphene oxide (NGO) was prepared by Hoffman rearrangement to make sure that the amino group grafted on GO directly. It was blended with polyacrylate directly without high-temperature curing or additional hot-pressing. The advantage of this method is that the process is simple and feasible, and graphene has no influence on the preparation of polyacrylate. The results showed that the performances of the polyacrylate/NGO composite were improved by simply blending and pH adjusting. We also [21] prepared a flexible and mechanically robust waterborne polyacrylate/graphene@polydopamine composite by establishing a tough interface through polydopamine modification and pH-triggered hydrogen bonding. By adjusting the initial pH value in the process of film formation, the electrostatic repulsion between graphene@polydopamine and polyacrylate changed to a hydrogen bond, so as to improve the mechanical properties. Waterborne polyacrylate material has been widely used as a coating agent [22], leather finish [23], biomaterial [24], etc. That means pH-induced interaction between polyacrylate and graphene will significantly change the proportion of the composites.

In the present work, polyacrylate with carboxyl, hydroxyl and acylamino groups was synthesized via emulsion polymerization by introducing the monomers AA, HEA and AM, marked as P(MMA-BA-AA), P(MMA-BA-HEA) and P(MMA-BA-AM), respectively. Polyacrylate with different functional groups will show different surface changes at different pH. The polyacrylate/NGO composite was prepared by mixing NGO with each of the three kinds of polyacrylate. The effects of different pH and NGO amounts on the properties of polyacrylate were studied. The interfacial interactions of polyacrylate with different functional groups and NGO were investigated via mechanical tests, zeta potential, thermogravimetric analysis and film form process detection.

## 2. Materials and Methods

### 2.1. Materials

Amino-functionalized graphene oxide (NGO) was prepared by amination of graphene oxide through Hoffman rearrangement. The typical synthesis can be found in our previous work [20]. Methyl methacrylate (MMA), n-butyl acrylate (BA), acrylic acid (AA), hydroxyethyl acrylate (HEA), acrylamide (AM), sodium dodecyl sulfate (SDS), polyethylene glycol-400 (PEG-400, molecular weight 380–430 g/mol), ammonia water (NH_3_•H_2_O, 25–28%, aq.) and ammonium persulfate (APS) were purchased from Sinopharm Chemical Reagent Co., Ltd. All chemicals were used as received without any further purification. Deionized water was used throughout the experiment.

### 2.2. Preparation of Polyacrylate with Different Functional Groups

**Scheme 1:**
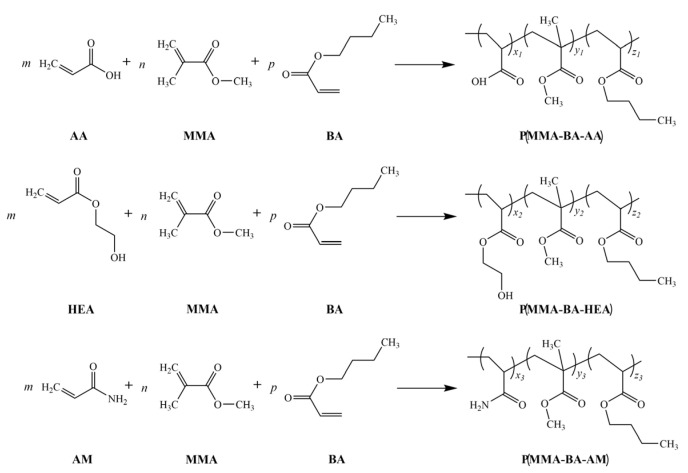
Schematic illustration for the preparation of polyacrylate.

Polyacrylate compounds containing different functional groups were obtained through emulsion polymerization of MMA, BA and functional monomers (AA, HEA or AM). The processes are presented in Scheme 1. Firstly, SDS (1.00 g), PEG-400 (0.25 g) and deionized water (71.00 g) were charged in a three-neck bottle with a thermometer, refluxing condensation pipe and stirrer. After the mixture was uniform, MMA (0.05 mol), BA (0.08 mol) and AA (0.12 mol) were added and stirred. Then, polymerization was carried out at 80 °C for 2 h with dropwise addition of a mixture of monomers (MMA (0.10 mol), BA (0.15 mol), AA (0.02 mol) and APS solution (30 g, 1.6%)). After cooling, polyacrylate materials containing different functional groups were obtained and defined as P(MMA-BA-AA), P(MMA-BA-HEA) and P(MMA-BA-AM). The amounts of AM (0.14 mol) and HEA (0.14 mol) were calculated to keep the molar weight the same as AA. That would keep the number of functional groups the same in different polyacrylate preparations.

### 2.3. Preparation of Polyacrylate/NGO Composites

The pH of P(MMA-BA-AA) was adjusted to different values (2, 4, 6, 7, 8) by adding ammonia hydroxide. Then, different amounts of NGO (0.1–0.7 wt%), in relation to polyacrylate, were added with the help of magnetic stirring (30 min, 100 rpm) and ultrasonic processing (30 min, 150 W) to obtain a uniform composite material. After that, P(MMA-BA-AA)/NGO was prepared. P(MMA-BA-HEA)/NGO and P(MMA-BA-AM)/NGO composite material were prepared by the same procedure.

Films were prepared by naturally drying 12 g of composite material at pH 7 in covered Petri dishes to prevent contamination. All dried films were placed in a desiccator for 24 h before the next procedures in order to keep moisture the same.

### 2.4. Characterization

For mechanical tests, the films were cut by a dumbbell-shaped sampler into 80 × 15 mm^2^ sizes. The bench markers used to measure elongation were 3051 mm^3^. The precise sizes of the specimens were measured before carrying out each test. The break strength and elongation at break were obtained using a Gotech AI-3000 Servo control tensile testing machine. Each kind of sample was examined three times. The structure of the samples was analyzed via Fourier transform infrared spectroscopy (FT-IR, Bruker Alpha VECTOR-22, GER). Malvern Zetasizer Nano-ZS (Malvern, UK) was used to measure the zeta potential of the polyacrylate material particles (the material was diluted to 1% in solid content) and NGO aqueous suspensions (0.1 mg/mL as described above). The thermal degradation of polyacrylate/NGO composites was investigated using a Q500 thermo-analyzer instrument (TA Instruments, DE, USA). Samples of ~5 mg were heated from 30 to 600 °C at a linear rate of 10 °C/min under nitrogen flow (50 mL/min). The samples were kept in a dryer before the thermogravimetric analysis. The effect of NGO on the polyacrylate film-forming process was measured by Horus Coatings Drying (France Formulaction Ltd, TSL, France) to characterize the kinetics. The polyacrylate material was dropped into a groove, which was 5 mm wide and 500 μm deep, at 27 °C and 22% RH. The groove was placed under a detector to monitor microstructural changes during the film formation process. A Hitachi S4800 scanning electron microscope (SEM, Tokyo, Japan) was used to observe the morphologies of polyacrylate/NGO composites.

## 3. Results and Discussion

### 3.1. Structural Analysis of Polyacrylate with Different Functional Groups

**Figure 1:**
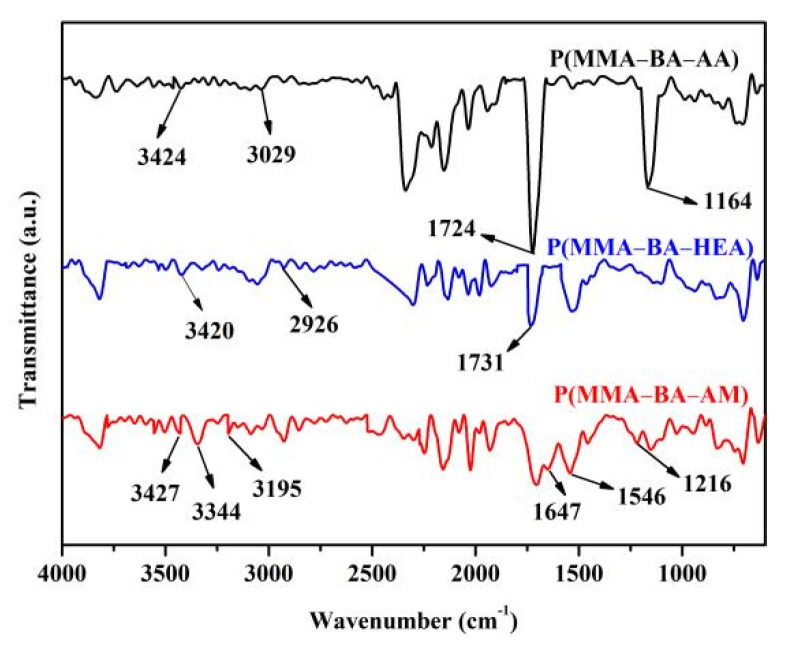
FT-IR spectra of P(MMA-BA-AA), P(MMA-BA-HEA) and P(MMA-BA-AM).

The goal of introducing AA, HEA and AM in the polyacrylate emulsion polymerization is to graft carboxyl, hydroxyl and acylamino groups on polyacrylate, respectively. The FT-IR spectra of P(MMA-BA-AA), P(MMA-BA-HEA) and P(MMA-BA-AM) are shown in Figure 1. As for the P(MMA-BA-AA), the absorption peaks that appear at 1164 cm^−1^ and 1724 cm^−1^ belong to the stretching vibration of C=O. The peaks at 3424 cm^−1^ and 3029 cm^−1^ belong to the stretching vibration of -OH. For P(MMA-BA-HEA), the band at 3420 cm^−1^ corresponds to the stretching vibration of -OH. The band at about 2926 cm^−1^ is assigned to C-H stretching vibration, while the band at 1731 cm^−1^ is attributed to the stretching vibration of C=O. For P(MMA-BA-AM), the peaks at 3427 cm^−1^, 3344 cm^−1^ and 3195 cm^−1^ are attributed to the stretching vibration of N-H. The band at 1647 cm^−1^ corresponds to the stretching vibration of C=O. The absorption peaks that appear at 1546 cm^−1^ and 1216 cm^−1^ belong to the stretching vibration of C-N. Therefore, polyacrylates with carboxyl, hydroxyl and acylamino groups were successfully prepared.

### 3.2. Stability of Polyacrylate/NGO Composite Material

The amino group in NGO as well as the carboxyl, hydroxyl and acylamino groups on the polyacrylate matrix are all functional groups and sensitive to pH. When mixing NGO with polyacrylate, pH acts as an important factor in stabilizing NGO in the polymer matrix. Therefore, stability is an important aspect to study in the interaction of NGO and polyacrylate. Table 1 shows the stability of polyacrylate/NGO composites at different pH.

**Figure 2:**
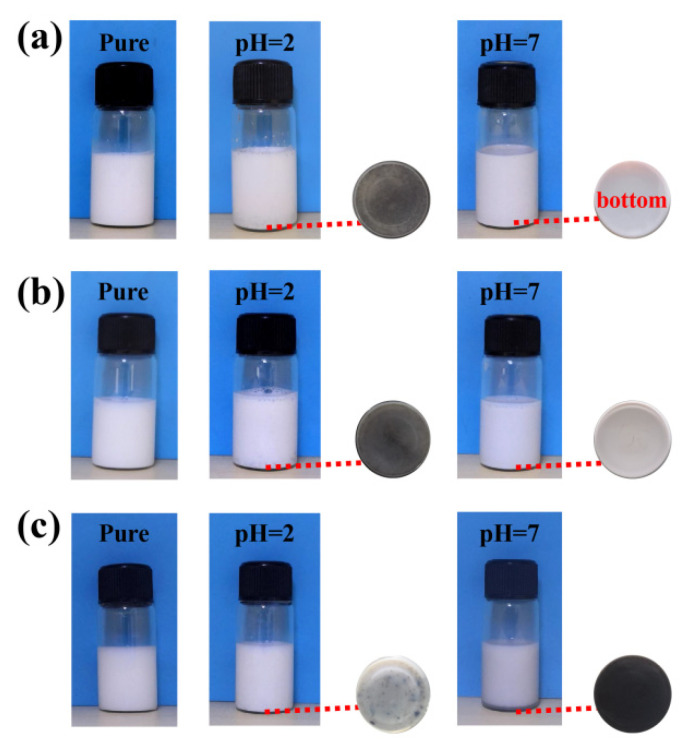
Digital camera photograph of polyacrylate/NGO composite materials at different pH. The circular photo corresponds to the bottom of the glass bottle: (**a**) P(MMA-BA-AA)/NGO; (**b**) P(MMA-BA-HEA)/NGO; (**c**) P(MMA-BA-AM)/NGO.

As shown in Table 1, the polyacrylate/NGO materials present different stabilities as a function of pH. The P(MMA-BA-AM)/NGO composite material demulsificated no matter if the pH value was high or low. When the pH value was 2 or 4, the addition of NGO demulsificated the P(MMA-BA-AA)/NGO and P(MMA-BA-HEA)/NGO materials with NGO deposits at the bottom of the glass bottle. With the increase in pH (higher than 6), the composites became stable (as shown in Figure 2). These phenomena indicate that the dispersion of NGO in the polyacrylate material heavily relies on pH.

**Figure 3:**
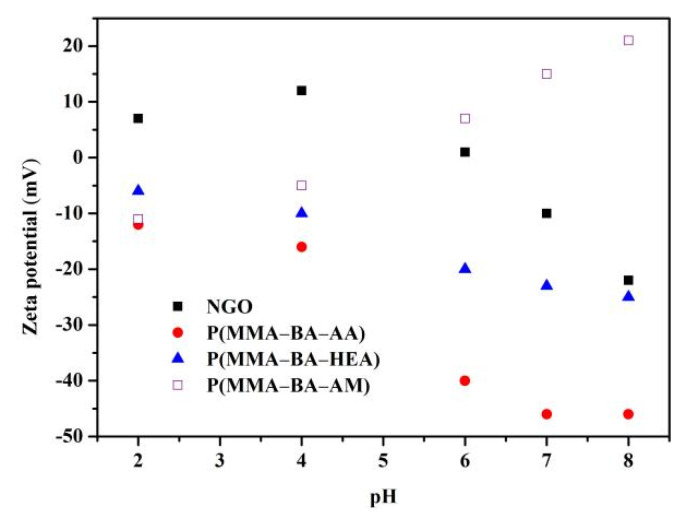
Effect of pH on the zeta potential of polyacrylate and NGO.

In order to figure out the reason of different stabilities at different pH values of the composite material, the zeta potentials of NGO and polyacrylate material particles were measured via dynamic light scattering (DLS), as shown in Figure 3. It was found that P(MMA-BA-AA), P(MMA-BA-HEA) and P(MMA-BA-AM) material particles were all negatively charged to a different degree when the pH value was less than 6, while NGO was positively charged at this pH range. That means the electrostatic attraction of the opposite charges led NGO to interact with polyacrylate and finally demulsificated the polyacrylate/NGO composite material.

**Scheme 2:**
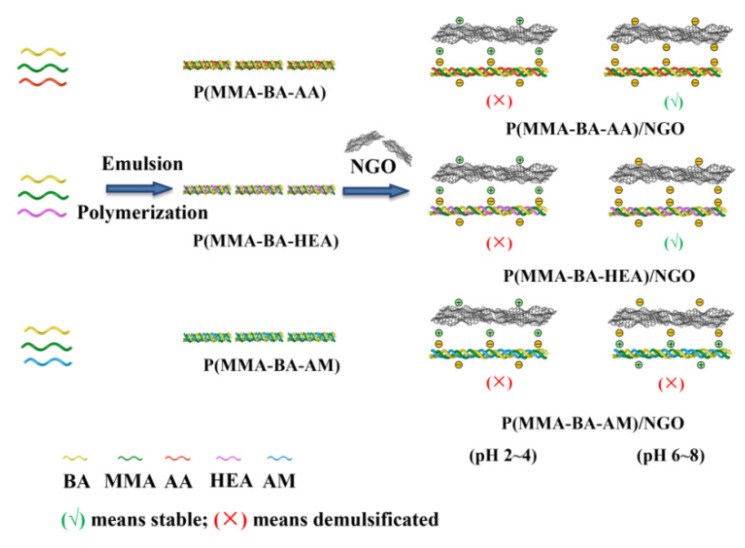
Schematic illustration for the stability of polyacrylate/NGO composites.

The stability of polyacrylate/NGO at different pH values is also clearly presented in Scheme 2. NGO was negatively charged when the pH value was greater than 6, as the previous work reported [20]. P(MMA-BA-AA) and P(MMA-BA-HEA) were equally negatively charged with NGO. Electrostatic repulsion between NGO and P(MMA-BA-AA) or P(MMA-BA-HEA) particles keeps the material stable and NGO finely dispersed. However, the synergistic effect between the -CONH_2_ group and SDS/PEG-400 on the surface of emulsion particles makes P(MMA-BA-AM) have opposite charges with NGO at any pH value. Therefore, there is electrostatic attraction between P(MMA-BA-AM) and NGO. For the emulsion, this electrostatic attraction is not conducive to emulsion stability. That means the P(MMA-BA-AM)/NGO composite material could not be stable within the whole range of measured pH values.

### 3.3. Mechanical and Thermal Properties of Polyacrylate/NGO Composite Material

The goal of incorporating NGO into polyacrylate material is to study the conditions of composite formation and figure out the interactions between the functional groups of NGO and polyacrylate. As described above, P(MMA-BA-AM)/NGO was not stable, so the data could not be presented.

**Figure 4:**
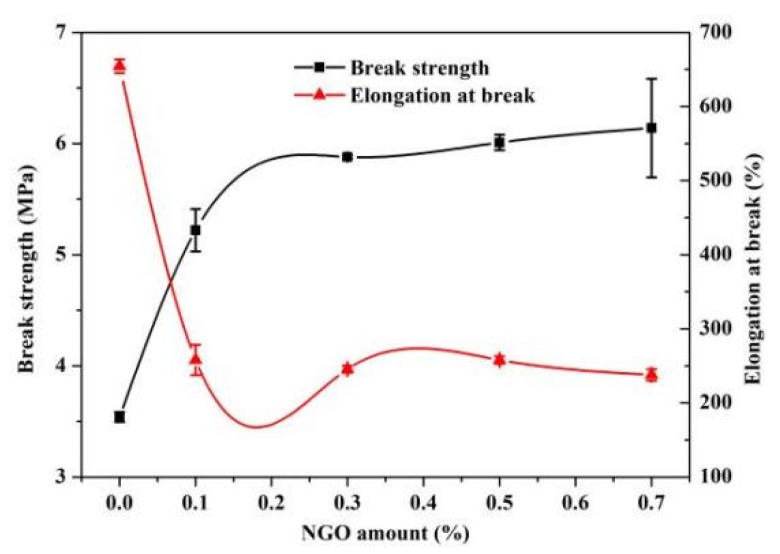
Effect of NGO amounts on mechanical properties of P(MMA-BA-AA)/NGO films.

When the pH of P(MMA-BA-AA) was adjusted to 7, the maximum amount of NGO that could be mixed into P(MMA-BA-AA) was 0.7%. The mechanical properties of P(MMA-BA-AA)/NGO films with NGO amounts are shown in Figure 4. As can be seen, the break strength and elongation at break of pure P(MMA-BA-AA) were about 3.54 MPa and 654.4%, respectively. After adding 0.1% of NGO, the break strength of P(MMA-BA-AA)/NGO increased to 5.22 MPa by 47%, and the elongation at break decreased to 257.9% by 61%. With the NGO amounts continuously increasing, the break strength increased to 6.14 MPa by 73%. However, the increased extent of elongation at break showed minimized change.

**Figure 5:**
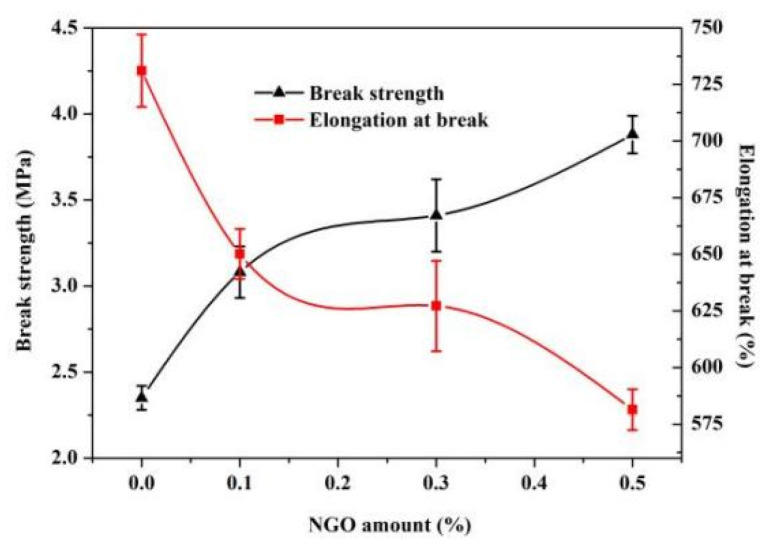
Effect of NGO amounts on mechanical properties of P(MMA-BA-HEA)/NGO films.

The situation of polyacrylate with the hydroxyl group, as P(MMA-BA-HEA), was different after adding NGO. When the pH of P(MMA-BA-HEA) was adjusted to 7, the maximum amount of NGO was 0.5%, as shown in Figure 5. More NGO would precipitate. The break strength and elongation at break of pure P(MMA-BA-HEA) were 2.35 MPa and 731.1%, respectively. After adding 0.1% of NGO, the break strength of P(MMA-BA-HEA)/NGO increased to 3.08 MPa by 31%, and the elongation at break decreased to 650.2% by 11%. With the NGO amounts increasing, the break strength increased to a maximum of 3.88 MPa by 65%, and elongation at break decreased to 581.4% by 20%.

As shown in the results above, polyacrylate with the carboxyl group had a stronger interaction with NGO than that of polyacrylate with the hydroxyl group. Firstly, the maximum NGO loading amounts for P(MMA-BA-AA)/NGO and P(MMA-BA-HEA)/NGO were 0.7% and 0.5%, respectively. Secondly, with the same NGO amount, the increasing rate of break strength of P(MMA-BA-AA)/NGO was larger than that of P(MMA-BA-HEA)/NGO, and the rate of decline elongation at break of P(MMA-BA-AA)/NGO was smaller. The increase in break strength was caused by the strengthening effect of graphene-based nanomaterials, as other researchers reported [25,26,27]. Briefly, the amino group on NGO would have a certain degree of interfacial interaction with carboxyl or hydroxyl groups on polyacrylate. The strength load could transfer from polyacrylate to NGO, so the break strength was reinforced. However, as a stratified material, NGO astricts the molecular entanglement and movement of polyacrylate. The elongation at break was weakened. As is known, the mechanical properties of polymer nanocomposites are significantly affected by the interfacial interaction between fillers and the polymer matrix [28]. As shown in the results, after the polyacrylate films were formed, NGO had a stronger effect with P(MMA-BA-AA) than P(MMA-BA-HEA). This may be caused by the interaction of functional groups of NGO and polyacrylate.

**Figure 6:**
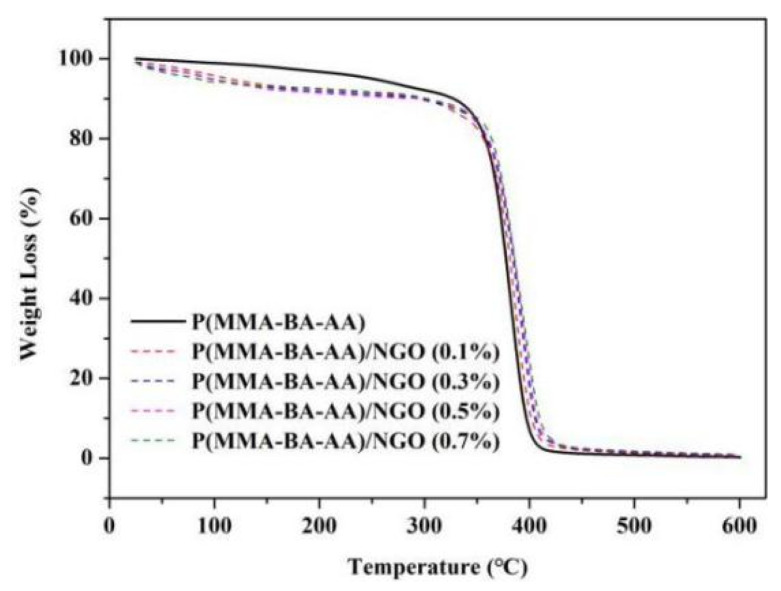
TGA curves of P(MMA-BA-AA)/NGO with different NGO amounts.

**Figure 7:**
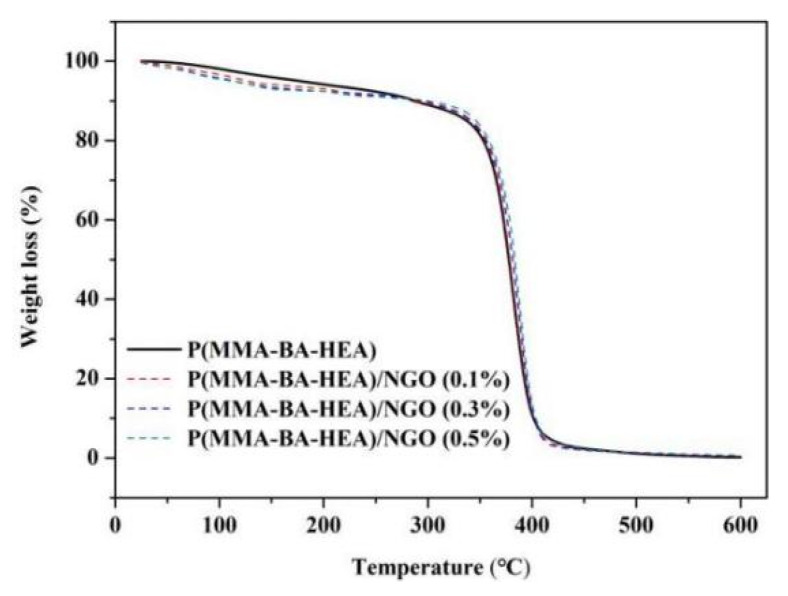
TGA curves of P(MMA-BA-HEA)/NGO with different NGO amounts.

Testing the change in thermal properties of polymer-based nanocomposites is an effective method to illustrate the interaction of polymers and nanomaterials [29]. Figure 6 and Figure 7 show the TGA curves of polyacrylate and polyacrylate/NGO.

According to the study of Xue [30], the thermal weightlessness of GO is divided into two main stages, that is, dehydration and decomposition of oxygen-containing groups. The first stage is observed from 25·°C to 160 °C with weight loss of 16% for the dehydration of GO. The second stage is observed from 160 °C to 240 °C with a rapid weight loss of 40% owing to the removal of the oxygen-containing groups. In Figure 6, pure P(MMA-BA-AA) shows one stage of thermal decomposition behavior. That means there was only one inflection point below 400 °C. The maximum decomposition temperature was 381.4 °C. When NGO was added, P(MMA-BA-AA)/NGO decomposed faster than P(MMA-BA-AA) below 150 °C. With the increase in NGO content, the decomposition rate was faster, which is related to the amount of adsorbed water released and the amount of NGO. After that, P(MMA-BA-AA)/NGO decomposed slower than P(MMA-BA-AA). As is known, P(MMA-BA-AA) is a hydrophilic polymer. The weight loss that occurred below 150 °C was associated with the thermal desorption of physically adsorbed and interlamellar water molecules. Because the carboxyl group of P(MMA-BA-AA) interacted with the amino group on NGO, the water loss at 30–150 °C for P(MMA-BA-AA)/NGO may be mostly attributed to physically adsorbed water, and that of P(MMA-BA-AA) was mostly attributed to interlamellar water. It indicated that water had a stronger binding force with P(MMA-BA-AA) than P(MMA-BA-AA)/NGO. That also proved the carboxyl group of P(MMA-BA-AA) interacted with the amino group on NGO. The maximum decomposition temperature of P(MMA-BA-AA)/NGO increased as NGO amounts rose. The situations of P(MMA-BA-HEA) and P(MMA-BA-HEA)/NGO were similar as those of P(MMA-BA-AA) and P(MMA-BA-AA)/NGO. Macrocosmically, NGO could increase the thermal properties of P(MMA-BA-HEA), and the maximum decomposition temperature of P(MMA-BA-HEA)/NGO increased as NGO amounts rise. However, the weight loss related to water occurred below 140 °C, and the weight loss extent was smaller than that of polyacrylate with the carboxyl group. In order to keep moisture uniform, all samples were placed in a desiccator for 24 h before the next procedures. Considering this from a thermal analysis perspective, the interaction between the amino group on NGO and carboxyl group of P(MMA-BA-AA) was stronger than that of the amino group on NGO and hydroxy group of P(MMA-BA-HEA).

**Figure 8:**
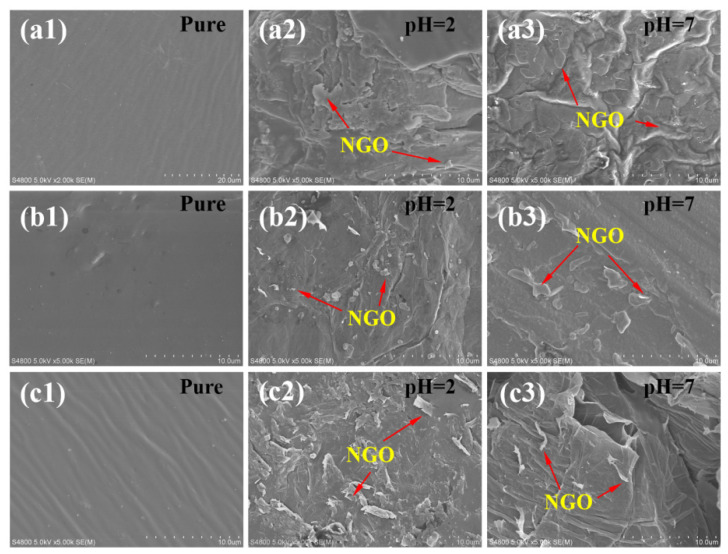
SEM images of (**a1**) P(MMA-BA-AA) film, P(MMA-BA-AA)/NGO dregs at pH 2 (**a2**) and film at pH 7 (**a3**); (**b1**) P(MMA-BA-HEA) film, P(MMA-BA-HEA)/NGO dregs at pH 2 (**b2**) and film at pH 7 (**b3**); (**c1**) P(MMA-BA-AM) film, P(MMA-BA-AM)/NGO dregs at pH 2 (**c2**) and pH 7 (**c3**).

The morphologies of polyacrylate and polyacrylate/NGO composites are compared in Figure 8. As can be seen from Figure 8 the surfaces of the pure polyacrylates were relatively flat and presented a typical state of polyacrylate emulsion film. When NGO was introduced, the surface of the composite film became rough (as shown in Figure 8(a3,b3)). Under the condition of pH 2, the composites were unstable and dregs appeared. Therefore, the dregs were also observed by SEM. It can be seen from Figure 8(a2,b2,c2) that NGO existed in the dregs, especially in P(MMA-BA-AM)/NGO (Figure 8(c2)). For P(MMA-BA-AM)/NGO, under the condition of pH 7, the composite material cannot be stable in the form of emulsion and would demulsificate. It can also be observed from Figure 8(c3) that NGO was large in size. The above results are consistent with the analysis of zeta potential described earlier.

**Figure 9:**
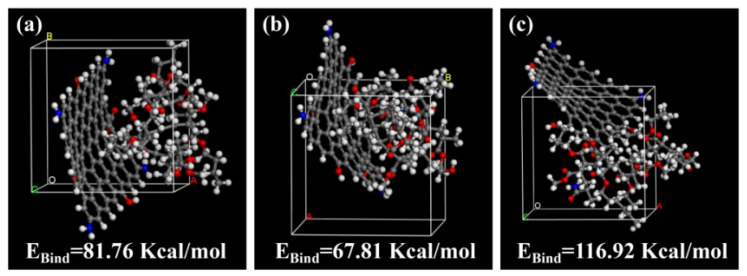
The molecular dynamics simulation of NGO and polymer chains: (**a**) P(MMA-BA-AA), (**b**) P(MMA-BA-HEA) and (**c**) P(MMA-BA-AM).

The mechanical properties of polymer nanocomposites are significantly affected by the interfacial interaction between the amino group on NGO and the carboxyl group or hydroxyl group on polyacrylate. To verify the existence of interactions between NGO and P(MMA-BA-AA), P(MMA-BA-HEA) and P(MMA-BA-AM), Materials Studio (MS) 8.0 was used to calculate the binding energy (EBind) between NGO and polymer chains. C_92_O_5_N_4_H_128_ was used as the model of NGO. The interaction energy (E_Inter_) between NGO and different polymer chains was calculated as follows:

$$E_{\mathrm{Inter}}=E_{\mathrm{Total}}-(E_{\mathrm{GNS}}-E_{\mathrm{Poly}})$$

where E_Total_ is the total energy of each system in equilibrium. E_GNS_ and E_Poly_ are single point energies of NGO and polymer chains, respectively. The negative value of E_Inter_ was defined as E_Bind_. As shown in Figure 9 , E_Bind_ for NGO on P(MMA-BA-AM) was calculated to be 116.92 kcal/mol, which is higher than that of NGO and P(MMA-BA-HEA) (67.81 kcal/mol) as well as NGO and P(MMA-BA-AA) (81.76 kcal/mol). The electrostatic repulsion between NGO and P(MMA-BA-AA) or P(MMA-BA-HEA) material particles keeps the material stable and NGO finely dispersed.

### 3.4. Influence of NGO on Polyacrylate/NGO Composite Film-Forming Processes

**Scheme 3:**
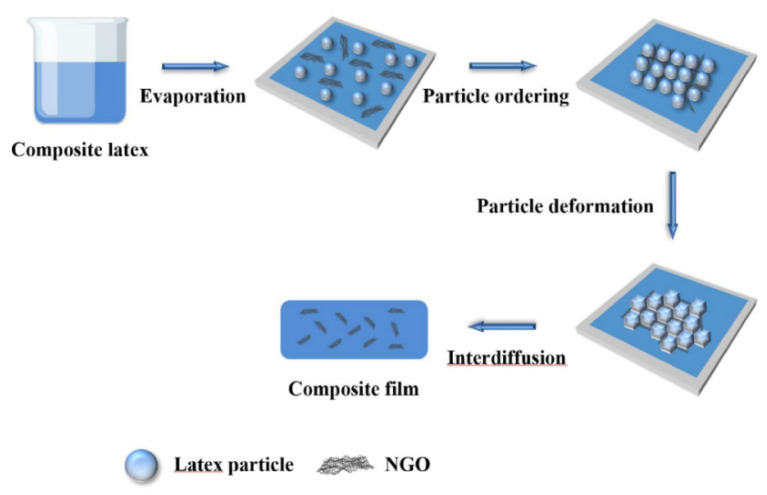
Schematic illustration for the film-forming process of polyacrylate/NGO composites.

**Figure 10:**
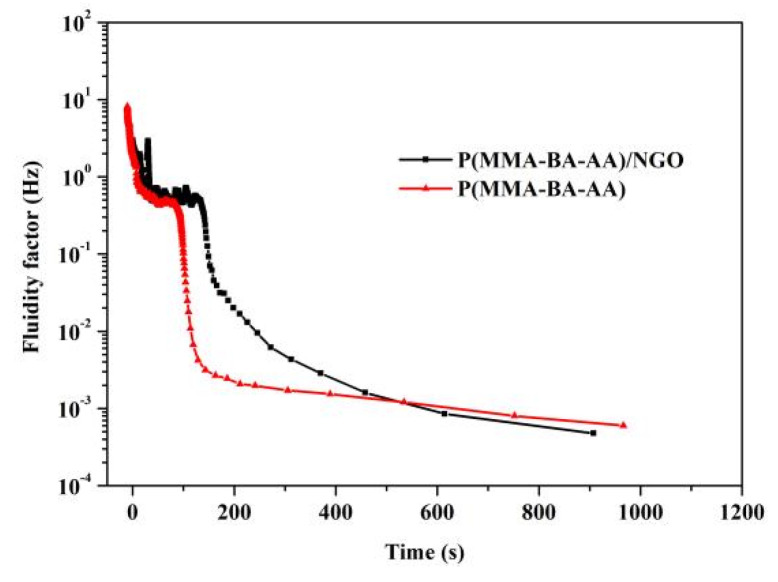
Kinetics of material film-forming processes for P(MMA-BA-AA) and P(MMA-BA-AA)/NGO.

**Figure 11:**
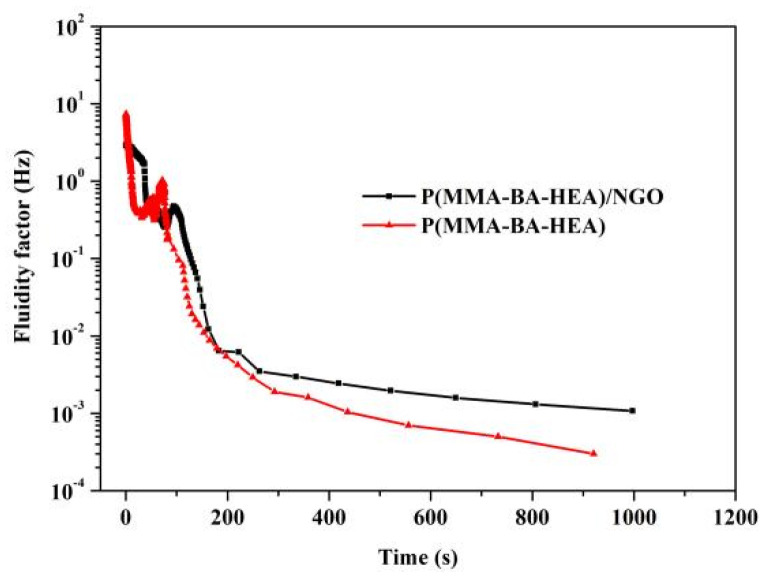
Kinetics of material film-forming processes for P(MMA-BA-HEA) and P(MMA-BA-HEA)/NGO.

The film-forming process plays a key role on the properties of polyacrylate nanocomposites. To explain the differences observed on the kinetics, the duration was divided into four stages necessary for the sample to turn to dry (Table 2): open time, dust free, set to touch and touch dry, as obtained automatically. In order to study the influence of NGO on the polyacrylate film-forming process, the process was measured by Horus Coatings Drying to characterize the kinetics. The results are shown in Figure 10 and Figure 11. Following Brun [31], the four stages represent the evaporation, particle ordering, particle deformation and interdiffusion of the material. If the evaporation time is too short, it means that the water vapor cannot evaporate sufficiently, which will affect the construction speed. If there is sufficient time in the particle ordering phase, the performance of the resulting film will be improved. The film-forming process of composite material is shown in Scheme 3.

It can be seen from Table 2 that, after NGO was added, evaporation time increased from 18 s to 34 s. The velocity of water evaporation slowed down because of the barrier effect of the NGO lamellar structure. The particle ordering time increased from 71 s to 98 s. With the evaporation of water and NH_3_•H_2_O, the interaction between the carboxyl group of P(MMA-BA-AA) and the amino group on NGO changed from electrostatic repulsion to electrostatic attraction. The interaction made the particle ordering time longer. After the relative position of P(MMA-BA-AA) and NGO was steady, the particle deformation time and interdiffusion time decreased by about 100 s. Firstly, movement of the P(MMA-BA-AA) molecular chain would be limited by NGO. Secondly, the water-retaining property of P(MMA-BA-AA) was weakened by the interaction of P(MMA-BA-AA) and NGO. Compared with P(MMA-BA-HEA), evaporation time of P(MMA-BA-HEA)/NGO increased from 21 s to 39 s. It was similar to P(MMA-BA-AA)/NGO. The particle ordering time increased from 60 s to 67 s. The increasing extent was not as much as that of P(MMA-BA-AA)/NGO. In addition, the particle deformation time and interdiffusion time decreased, but the rate of change was lower for P(MMA-BA-AA)/NGO. It proved that the interaction degrees of the functional groups for the two polyacrylate/NGO composites were different. During the film-forming process, the interaction between P(MMA-BA-AA) and NGO was stronger than that of P(MMA-BA-HEA) and NGO.

## 4. Conclusions

Polyacrylate compounds with different functional groups were prepared by introduction of AA, HEA and AM. When NGO was added, pH had a very important effect on the stability of polyacrylate/NGO composites because different functional groups such as carboxyl, hydroxyl, acylamino and amino groups charged the polyacrylate and NGO differently. Furthermore, it influenced the properties of the polyacrylate film. P(MMA-BA-AM)/NGO was not steady at any pH. P(MMA-BA-AA)/NGO and P(MMA-BA-HEA)/NGO were steady when the pH was greater than 6. NGO could toughen both P(MMA-BA-AA) and P(MMA-BA-HEA), but the effect of P(MMA-BA-AA)/NGO was better than P(MMA-BA-HEA)/NGO. Combined with the TGA results, the interaction between the amino group on NGO and carboxyl group of P(MMA-BA-AA) was stronger than that of the amino group on NGO and hydroxy of P(MMA-BA-HEA). The kinetics study indicated that NGO influenced polyacrylate through the whole film-forming process.

## Figures and Tables

**Table 1:** Stability of polyacrylate/NGO composite materials at different pH.

pH Value	2.0	4.0	6.0	7.0	8.0
P(MMA-BA-AA)/NGO	×	×	√	√	√
P(MMA-BA-HEA)/NGO	×	×	√	√	√
P(MMA-BA-AM)/NGO	×	×	×	×	×

√ means stable, × means demulsificated.

**Table 2:** The duration of stages for polyacrylate and polyacrylate/NGO material turn to dry.

Samples	Open Time (s)	Dust Free (s)	Set to Touch (s)	Touch Dry (s)
P(MMA-BA-AA)	18	71	233	667
P(MMA-BA-AA)/NGO	34	98	134	575
P(MMA-BA-HEA)	21	60	130	714
P(MMA-BA-HEA)/NGO	39	67	112	608

## Data Availability

The data presented in this study are available on request from the corresponding author.
